# Case report of extranodal NK/T-cell lymphoma mimicking Behcet syndrome

**DOI:** 10.1097/MD.0000000000044329

**Published:** 2025-09-05

**Authors:** Songyi Park, Jeemin Yim, Hye Eun Park, Jin Hyun Park, Hyun-Sun Yoon, Jin Soo Kim, In Sil Choi, Young A. Kim, Ji Eun Kim, Eun Youn Roh, Ki Hwan Kim

**Affiliations:** a Department of Internal Medicine, Seoul Metropolitan Government Seoul National University Boramae Medical Center, Seoul, Republic of Korea; b Department of Pathology, Seoul Metropolitan Government Seoul National University Boramae Medical Center, Seoul, Republic of Korea; c Department of Dermatology, Seoul Metropolitan Government Seoul National University Boramae Medical Center, Seoul, Republic of Korea; d Department of Laboratory Medicine, Seoul Metropolitan Government Seoul National University Boramae Medical Center, Seoul, Republic of Korea.

**Keywords:** Behcet syndrome, extranodal NK/T-cell lymphoma

## Abstract

**Rationale:**

Extranodal natural killer (NK)/T-cell lymphoma is an uncommon non-Hodgkin lymphoma, prevalent in Asia. It often involves the nasal and upper airway regions but can disseminate to other sites like skin, soft tissue, testis, and gastrointestinal tract, characterized by Epstein–Barr virus association.

**Patient concerns:**

This report discusses a 48-year-old male initially diagnosed with Behcet syndrome with dry mouth, uveitis, pruritic macules, and human leukocyte antigen-B51 positivity. Despite steroid treatment, a growing testicular mass and erythema nodosum led to reevaluation.

**Diagnoses:**

Biopsy confirmed Epstein–Barr virus-positive extranodal NK/T-cell lymphoma. The imaging indicated lymphoma spread to the nasal cavity, lymphatic chains, bones, and spleen.

**Interventions:**

The patient received multi-agent SMILE (methotrexate, ifosfamide, L-asparaginase, mesna, etoposide, dexamethasone) chemotherapy.

**Outcomes:**

The patient achieved a metabolic complete response after 2 cycles of SMILE chemotherapy.

**Lessons:**

This report highlights the diagnostic pitfalls due to symptoms and signs overlapping between Behcet syndrome and extranodal NK/T-cell lymphoma.

## 1. Introduction

Extranodal natural killer (NK)/T-cell lymphoma is an uncommon type of lymphoma, more frequently reported in Asia than in Western countries.^[1]^ It typically involves the nasal and upper airway regions but can also affect other areas such as the skin, soft tissue, testis, upper respiratory tract, and gastrointestinal tract. The disease is characterized by local invasion and necrosis with Epstein–Barr virus (EBV) infection of the neoplastic cells.^[2–4]^ The standard treatment for NK/T-cell lymphoma combines chemotherapy and radiotherapy, particularly for localized nasal forms, achieving overall response rates of 81% to 96% in studies. Concurrent or sequential chemotherapy with radiotherapy is preferred, as isolated radiotherapy sometimes shows systemic failure. Regimens including l-asparaginase yield complete response rates of 61% to 81%, especially in advanced stages.^[5]^ Early-stage nasal lymphomas have a 5-year overall survival rate of around 70%, while advanced NK-cell leukemia is associated with poor survival rates of about 40% at 2 years.^[6]^

Although extranodal NK/T-cell lymphoma has defined clinical and therapeutic characteristics, it is important to recognize that other disorders with similar mucocutaneous features, such as Behcet syndrome, may sometimes mimic these neoplastic conditions. Behcet syndrome is an inflammatory disorder, characterized by recurrent oral aphthous ulcers, genital ulcers, ocular disease like uveitis, and skin lesions.^[7]^ The human leukocyte antigen (HLA)-B51 allele is well known to be associated with Behcet syndrome.^[8]^

This case was initially diagnosed as Behcet syndrome due to dry mouth, uveitis, pruritic macules, and genetic results. However, the patient was re-diagnosed with extranodal NK/T-cell lymphoma upon biopsy of a testicular mass and skin lesion. We report this case as extranodal NK/T-cell lymphoma disguised as Behcet syndrome.

## 2. Case description

A 48-year-old man presented to our department with palpable growing testicular mass.

The patient had no notable medical history other than hypertension. About 4 months ago, he contracted COVID-19, after which his joint pain worsened, prompting him to visit another hospital. Along with the worsening joint symptoms, he developed cough, rhinorrhea, nasal obstruction, sore throat, and decreased vision. Additionally, approximately 2 months prior to his current presentation, a large, pruritic macule appeared on his skin. Based on these symptoms, he visited another hospital where various tests were conducted. The results showed negative rheumatoid factor, anti-citrullinated protein antibodies, antinuclear antibody, anti-Ro/La antibody, HLA-B27, and antineutrophil cytoplasmic antibody, but HLA-B51 was positive. Additionally, ophthalmologic examination revealed uveitis. Based on the clinical presentation and laboratory findings, the other hospital diagnosed him with Behcet syndrome.

**Figure 1.**
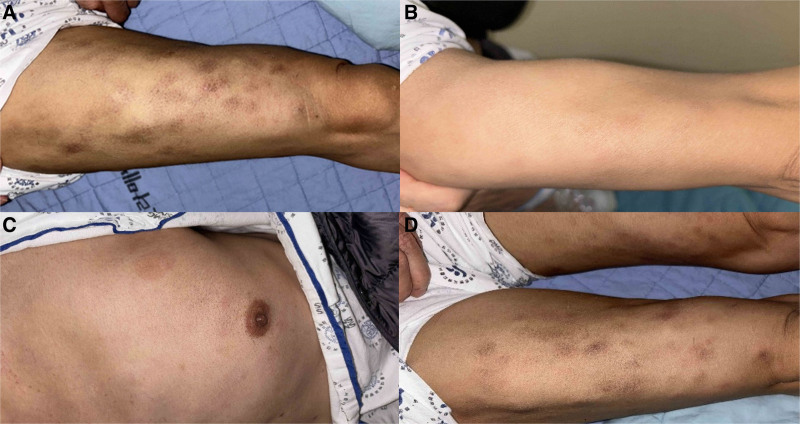
Large pruritic macules on the skin. (A) Right thigh. (B) Right upper arm. (C) Left chest wall. (D) Both thigh.

**Figure 2.**
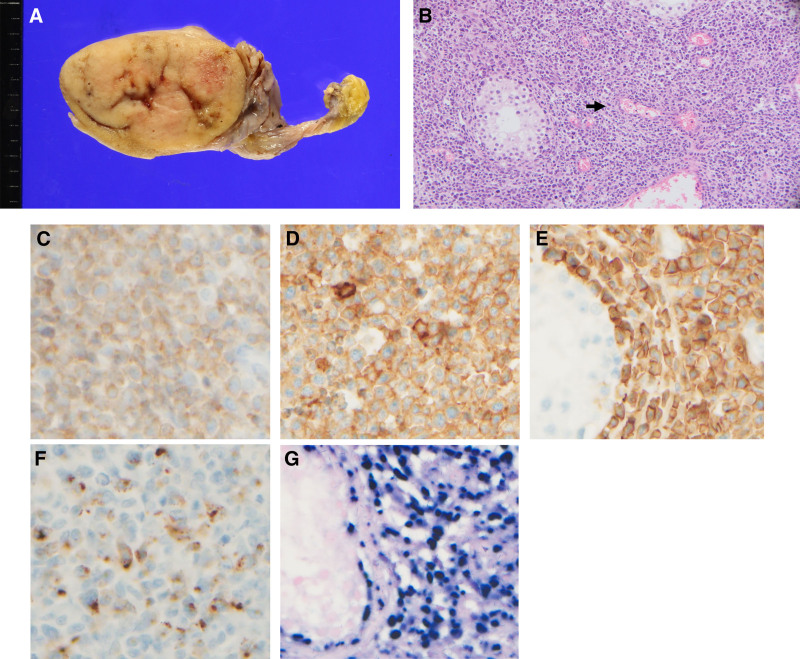
Pathology of right testicle. (A) The resected testis showed yellow-tan gray colored solid mass measuring about 6 cm. (B) Microscopically, diffuse infiltration of atypical lymphoid cells with angio-destructive lesions (arrow) was sufficient to suspect malignant lymphoma. (C) CD3 stain. (D) CD43 stain. (E) CD56 stain. (F) Granzyme stain (all × 400). The tumor cells were positive for CD3, CD43, CD56 and granzyme but negative for CD4, CD5, CD8, and CD20 by immunohistochemistry. (G) Epstein-Barr virus-encoded ribonucleic acid in situ hybridization revealed almost all atypical lymphocytes were positive for EBV (hematoxylin eosin, x 50, inset: EBER in situ hybridization, x 400).

The other hospital physician started prednisolone at 20 mg once daily. After approximately 1 month of treatment, the joint pain showed signs of improvement, but symptoms of dry mouth and dry eyes persisted. Additionally, erythema nodosum appeared as a new manifestation (Fig. 1), and the testicular mass gradually enlarged, prompting the patient to seek care at our hospital. To evaluate the testicular condition, we conducted contrast-enhanced computed tomography. The imaging revealed an enlarged right testicle, raising suspicion for possible testicular cancer; however, there was no evidence of metastasis. Thus, we performed a right radical orchiectomy for the palpable and tender testicular mass, which confirmed the diagnosis (Fig. 2). Pathology of the right testicular mass showed typical EBV-positive extranodal NK/T-cell lymphoma.

**Figure 3.**
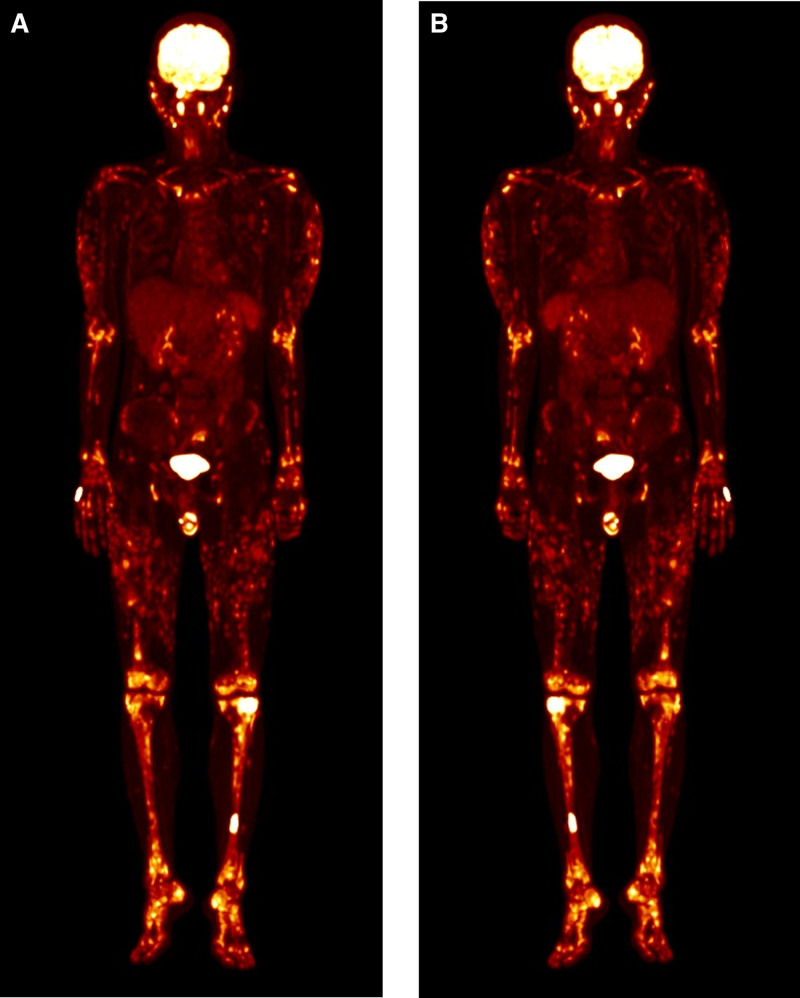
FDG-PET scan. (A) Front. (B) Back. FDG-PET scan showed probable lymphoma involvement including bilateral nasal cavity and left ethmoid sinus, multiple lymphatic chains both sides of the diaphragm, subcutaneous lesions, bones, and spleen. FDG-PET = fluorodeoxyglucose-positron emission tomography.

On fluorodeoxyglucose (FDG)-positron emission tomography scan from skull to foot for staging work up, probable lymphoma involvement was identified in the bilateral nasal cavity and left ethmoid sinus, multiple lymphatic chains on both sides of the diaphragm, subcutaneous lesions, bone, and spleen (Fig. 3).

**Figure 4.**
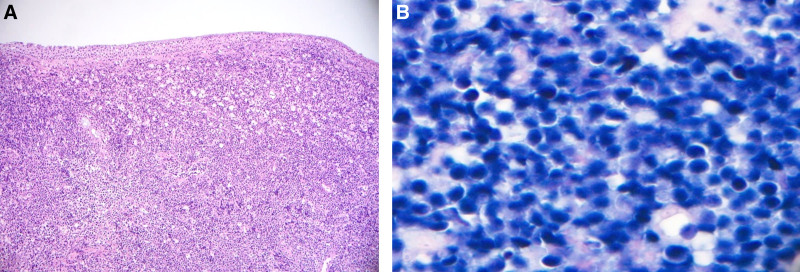
(A) Hematoxylin eosin (B) Epstein-Barr virus-encoded ribonucleic acid in situ hybridization. Histopathologic examination of the nasal mass showed diffuse infiltration of atypical lymphoid cells which were consistent with EBV-positive NK/T-cell lymphoma (hematoxylin eosin, x 50, inset: EBER in situ hybridization, x 400). EBV = Epstein–Barr virus, NK = natural killer.

Biopsies of the nasal cavity mass and a bone marrow also verified lymphoma involvement (Fig. 4). Nasal biopsy revealed dense atypical lymphocytic infiltrates similar to those seen in the testicular lesion and EBV-positive cells in the bone marrow. Additionally, because the patient was initially diagnosed as Behcet syndrome based on skin macules, we performed a skin biopsy to differentiate the diagnosis. The pathology of the skin biopsy was also consistent with EBV-positive extranodal NK/T-cell lymphoma involvement.

**Figure 5.**
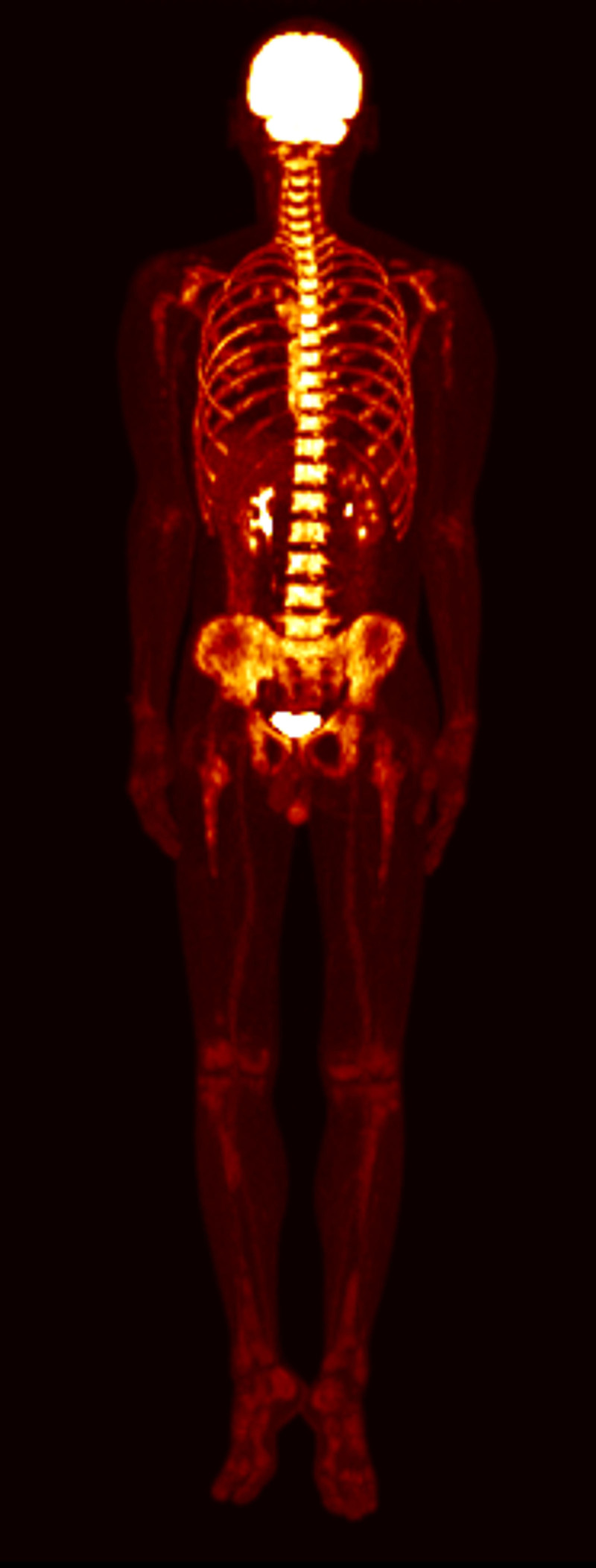
Follow-up FDG-PET Scan. No residual lesions in body. Probable reactive bone marrow change. FDG-PET = fluorodeoxyglucose-positron emission tomography.

Based on the pathology and imaging, we diagnosed the patient with stage IV EBV-positive extranodal NK/T-cell lymphoma, involving nasal cavity, multiple lymphatic chains, testicle, spleen, skin, and bone marrow. We initiated chemotherapy including methotrexate, ifosfamide, mesna, etoposide, dexamethasone, L-asparaginase (SMILE protocol).^[9]^ After 2 cycles of chemotherapy the patient showed a metabolic complete response on a follow-up FDG-PET scan without any side effects (Fig. 5).

## 3. Discussion

This case highlights the significant diagnostic challenge when extranodal NK/T-cell lymphoma, nasal type, mimics or coexists with Behcet syndrome. Both conditions can present with overlapping clinical features such as oral ulcers, skin lesions, and systemic symptoms, which often lead to initial misdiagnosis. Several case reports have documented instances where patients with persistent skin ulcers or lesions initially suspected to be Behcet disease were eventually diagnosed with lymphoma after treatment failure and subsequent tissue biopsies.^[10–12]^ Additionally, there are rare cases of concurrent Behcet syndrome and lymphoma, further complicating the clinical picture.^[13]^ These examples underscore how disease masking or association can hinder early diagnosis and appropriate management.

The overlapping clinical features between autoimmune or inflammatory conditions like Behcet syndrome and lymphoid malignancies such as NK/T-cell lymphoma are well recognized, partly because both involve immune dysregulation and inflammatory pathways. From a theoretical standpoint, this similarity is understandable, given that systemic inflammatory responses and immune activation can sometimes blur the differentiation between benign autoimmune processes and underlying malignancies.^[14]^

Clinical reports and studies have highlighted that such overlaps can often lead to diagnostic challenges. Many cases demonstrate that lymphoma may initially mimic autoimmune or inflammatory diseases, presenting with symptoms like oral or skin ulcers and systemic manifestations. When these symptoms do not respond to standard treatments, such cases underscore the importance of maintaining a broad differential diagnosis and considering malignancy, especially in recurrent or refractory cases. Repeated biopsies and thorough evaluation are often necessary to distinguish between these entities and avoid misdiagnosis, ultimately facilitating timely and appropriate management. These insights reinforce the need for clinicians to be vigilant about the potential for lymphomas to masquerade as autoimmune disorders, particularly when clinical responses deviate from expectations.^[15–17]^

The underlying reason for this masking often lies in the shared inflammatory and immune-related manifestations of both diseases, despite their different pathophysiological mechanisms. Their similar clinical presentations can lead clinicians astray, particularly when symptoms become atypical or refractory to standard treatments. Understanding these overlapping features is essential for avoiding diagnostic delays.

Extranodal NK/T-cell lymphoma, nasal type, is an aggressive non-Hodgkin lymphoma derived from NK or cytotoxic T cells. It most commonly presents in the upper aerodigestive tract, particularly the nasal and nasopharyngeal regions. Clinically, patients may exhibit symptoms such as nasal obstruction, epistaxis, nasal discharge, and facial swelling.^[1]^ Histologically, it demonstrates angiocentric and angio-destructive growth patterns, often with necrosis, and is associated with EBV infection. The disease progression is typically aggressive, with local invasion and dissemination being common.^[1–3]^

On the other hand, Behcet syndrome is a chronic, relapsing, multisystem inflammatory disorder characterized by oral and genital ulcerations, skin lesions, and uveitis. Its exact etiology remains unclear, but it is believed to involve an autoimmune response triggered by environmental factors in genetically predisposed individuals. Clinically, patients with Behcet syndrome may present with recurrent oral and genital ulcers, uveitis, skin lesions, and arthritis.^[7]^ Since these symptoms overlap significantly with those of lymphoma, the diagnostic process can be complicated by the similarities.

In our case, a patient initially presented with features suggestive of Behcet syndrome, including HLA-B27 positivity, a transient positive response to steroid therapy, and the absence of testicular swelling. However, subsequent pathology revealed extranodal NK/T-cell lymphoma involving multiple sites, including the testis, nasal mass, bone marrow, and skin. This unexpected diagnosis underscores the importance of a comprehensive evaluation and the limitations of relying solely on clinical features. The case is reminiscent of others reported in the literature, where patients with presumed Behcet disease, particularly those with persistent or atypical skin ulcers, were diagnosed with lymphoma after treatment failure and tissue biopsy.^[10–12]^ Some reports also highlight cases where lymphoma co-occurs with Behcet, further complicating diagnosis.^[13]^

HLA B27 positivity in the setting of lymphoma is a rare finding and may add complexity to the diagnostic process by mimicking rheumatologic conditions such as Behcet syndrome.^[18,19]^ Additionally, the initial response to steroids might transiently improve lymphoproliferative symptoms, further shielding the underlying malignancy. The absence of testicular swelling at presentation added an extra layer of difficulty, emphasizing the need for clinicians to maintain a high index of suspicion for atypical disease manifestations.

Ultimately, the confirmation of lymphoma involving multiple sites emphasizes its systemic and aggressive nature. Awareness of these overlapping features and the importance of considering a wide differential diagnosis, supported by evidence from published case reports and theoretical understanding, are essential in preventing diagnostic delays and ensuring timely, appropriate treatment.

## 4. Conclusion

In conclusion, this case serves as a reminder of the complexities inherent in diagnosing malignancies with overlapping clinical features with autoimmune conditions. The atypical presentation of extranodal NK/T cell lymphoma underscores the importance of considering a broad differential diagnosis and utilizing a multidisciplinary approach to ensure timely and accurate management in challenging clinical scenarios.

## Author Contributions

**Conceptualization:** Ki Hwan Kim.

**Data curation:** Jeemin Yim, Hye Eun Park.

**Investigation:** Hyun-Sun Yoon.

**Resources:** Jeemin Yim, Hye Eun Park, Hyun-Sun Yoon, Young A. Kim, Ji Eun Kim, Eun Youn Roh.

**Supervision:** Jin Hyun Park, Jin Soo Kim, In Sil Choi, Ki Hwan Kim.

**Validation:** Young A. Kim, Ji Eun Kim.

**Writing – original draft:** Songyi Park.

**Writing – review & editing:** Ki Hwan Kim.
